# Pre-exercise and acute movement-evoked pain trajectories during a 24-week outdoor walking program for knee osteoarthritis (WALK)

**DOI:** 10.1016/j.ocarto.2024.100481

**Published:** 2024-05-14

**Authors:** S.J.J. Drummen, D. Aitken, S. Balogun, K.L. Bennell, R.S. Hinman, M.L. Callisaya, P. Otahal, L. Blizzard, B. Antony, I.P. Munugoda, T. Winzenberg, G. Jones, L.E.J.M. Scheepers

**Affiliations:** a University of Tasmania, Hobart, Australia; b Australian National University, Canberra, Australia; c Monash University, Melbourne, Australia; d The University of Melbourne, Melbourne, Australia

**Keywords:** Aerobic, Exercise, OA, Pain, Flares, Rehabilitation

## Abstract

**Objectives:**

Exploring (1) pre-exercise and acute movement-evoked pain (AMEP) during an outdoor walking program in individuals with knee osteoarthritis (OA); and (2) comparing baseline physical performance and AMEP flares initiated by walking between participants with either a higher or lower attendance rate.

**Methods:**

Individuals with knee OA were prescribed a 24-week walking program, including one unsupervised walk and two supervised walk classes per week. Participants self-reported knee pain on a numerical rating scale (NRS; 0–10) before and after each supervised class. Mixed-effects models were used to investigate trajectories over time for pre-exercise pain and AMEP change (post-minus pre-exercise pain; positive value indicates flare-up). Baseline physical performance (6 tests) and AMEP flares were compared between participants with higher (attending ≥70% of supervised classes) and lower attendance rates.

**Results:**

Of 24 participants commencing the program, 7 (29%) withdrew. Over 24 weeks, pre-exercise pain improved by 1.20 NRS (95% CI -1.41 to −0.99), with estimated largest per class improvements during the first 8 weeks (−0.05 (−0.06 to −0.03) and plateauing around 20-weeks. The AMEP was estimated to improve by 0.19 NRS (95% CI -0.38 to −0.004) over 24-weeks, with improvements plateauing around 12-weeks. Participants with lower attendance (n ​= ​11) scored poorer on all physical performance tests and experienced a slight increase in AMEP during the first two weeks of the program.

**Conclusions:**

Participants improved in pre-exercise pain and AMEP in the first 20 and 12 weeks, respectively. Despite supervision, physical performance and AMEP flares may have contributed to lower attendance.

**Trial registration number:**

12618001097235.

## 1 Introduction

Therapeutic exercise is recommended as a first-line treatment of knee osteoarthritis (OA) [1]. However, there is a gap in the implementation of this recommendation [2,3]. The current treatment of knee OA predominantly involves medications and referral to orthopedic surgeons, rather than therapeutic exercise [2,3]. The reasons for the lack of exercise uptake are complex but may include clinician and patient misconceptions that OA is a wear-and-tear disease and that progression to joint replacement surgery is inevitable [4,5]. Consequently, patients may avoid movements that are painful, fearing that these will cause an exacerbation in symptoms [[6], [7], [8]].

The experience of an acute movement-evoked pain (AMEP) flare, defined as an increase in pain during and shortly after movement, is suggested to be a major and overlooked barrier to exercise adherence [[9], [10], [11]]. Knowledge about how AMEP from exercise changes over the course of an exercise program is important, as it would help clinicians and patients to manage expectations [8] and to tailor exercise accordingly. Thus far, only two studies have measured a trajectory in AMEP changes at each session of an exercise program for participants with knee OA symptoms [7,8]. In both studies AMEP changes fluctuated, but generally improved over the course of a neuromuscular exercise (NEMEX) program over 8–12 weeks [7,8]. Despite aerobic walking being highly recommended for individuals with knee OA, an AMEP trajectory during a walking intervention has not yet been investigated.

Several studies have demonstrated that consistent participation in exercise interventions results in better knee OA related health outcomes [12]. Supervision by a qualified healthcare professional can help to overcome barriers that individuals with knee OA may experience to therapeutic exercise [13]. For example, a healthcare professional is recommended to conduct a comprehensive physical assessment to determine movements that aggravate pain and use this information to tailor an exercise program to an individual [14]. Particularly at the initial stage of an exercise program, the dosage of exercise should be appropriate for the patient's physical ability [14].

In a recent pilot study from our group, we conducted a 24-week intensively supervised outdoor aerobic walking program for knee OA (WALK) [15]. We showed program adherence of 70% [15]. This was deemed acceptable, although greater adherence would be preferable to investigate the effects of the WALK program on knee OA symptoms and joint structure [15]. Therefore, the objectives of this ancillary study were to explore 1) trajectories in pre-exercise knee pain and in AMEP changes that occurred at each supervised class of the WALK program; and 2) walking class attendance in relation to initial AMEP flares and baseline physical performance.

## 2 Methods

This is an exploratory study ancillary to WALK, a pilot randomized controlled trial (RCT) that compared aerobic walking to usual care for knee OA [15]. The present study only includes those who were randomized to the aerobic walking group. The trial was registered on the Australian New Zealand Clinical Trials Registry prior to recruitment (12618001097235). Ethics approval was from the Tasmanian Health and Medical Human Research Ethics Committee (H0017108) and all participants provided written informed consent.

### 2.1 Recruitment and screening

Recruitment and screening took place from October 2018 to June 2019. Participants were recruited from the community with advertising through local and social media. Potential participants were pre-screened via telephone by a research assistant. Subsequently, face-to-face screening was performed, during which a detailed explanation of the project was given and written informed consent obtained. The inclusion and exclusion criteria are outlined in Table 1. Participants with conditions that precluded safe participation in exercise (e.g. a heart condition), as assessed by the Adult Pre-Exercise Screening Tool (stage 1) [16], were required to receive medical clearance from their general practitioner before being enrolled. When a participant had two eligible knees, the knee with the worst pain was selected as the study knee.

**Table 1:** Inclusion and exclusion criteria of participant selection.

Inclusion criteria	Exclusion criteria
- Aged 45 years or over - Knee BML present on MRI - ACR criteria for clinical knee OA - Symptomatic knee OA for at least 24 weeks with a VAS score of at least 40 ​mm/100 ​mm over the last 7 days - No difficulty in walking a city block - Willing and able to participate in a 24-week walking program	- Severe knee pain (>80 ​mm/100 VAS) - Currently meeting the guidelines for MVPA per week (>150 ​min/week) - Walking >10,000 steps/day - Other forms of arthritis - Undergone significant trauma to the ‘study’ knee in the previous 12 months - Received intra-articular therapy (e.g. corticosteroids, hyaluronic acid) in the study knee in the last 24 weeks - Anticipated need for knee or hip surgery within the following 24 weeks - Contra-indication to MRI - Planning to commence exercise or another new treatment for knee OA in the next 24 weeks - Using a gait aid - Unable to provide informed consent

ACR ​= ​The American College of Rheumatology; BML ​= ​bone marrow lesion; MRI ​= ​magnetic resonance imaging; MVPA ​= ​moderate to vigorous physical activity; VAS ​= ​visual analogue scale.

#### 2.1.1 Walking intervention

The participants were asked to complete three aerobic walking sessions per week for 24 weeks. Each week consisted of two walk classes in a supervised group of up to 10 participants and one unsupervised walk session at a location of their choice. The walking program was based on a protocol published by Ettinger et al. [17]. Each class lasted 1 ​h and consisted of a 10 ​min warm up [15], a 40 ​min walk aimed at 50–70% of heart rate reserve (using the validated Borg Rating of Perceived Exertion (RPE) Scale) [18], and a 10-min cool down consisting of slow walking and 3 flexibility exercises [15]. The supervised classes were each led by one supervisor who was either a physiotherapist or exercise physiologist. All study staff (supervisors and research assistants) participated in a half day workshop for protocol training including instructions about administering the program, monitoring attendance and pain, and receiving education about behavioral change [19]. Additionally, the supervisors attended an on-site introduction to the three outdoor class locations where optional walking back, early opt-out and loop options were shown. By using these options, the intensity and/or distance of the class could be tailored to each participant. At the supervised classes, adequate exercise intensity was guided by supervisors who used the RPE scale. Supervisors documented attendance and pain at the classes (two times/week, total 48). Attendance ≥70% was considered high attendance whereas attendance <70% was considered low attendance. Participants received an online questionnaire by email each week in which they were asked to report the completion of unsupervised (home) walking sessions, for which pain was not measured. The online questionnaire also gave participants the opportunity to provide feedback. To encourage class attendance, participants had flexibility to choose the day/time/location/supervisor, were allowed to bring family members on classes, and received a Fitbit upon enrolment. In addition, the intervention included several behavioral change techniques including social support, positive reinforcement, goal setting, rewards for attendance, frequent contact, and recognition in study update newsletters. Furthermore, participants received generic information about OA, community services and resources, and were asked not to start any new forms of exercise during the study period. If participants withdrew from the study before 24 weeks, the reason and date were recorded.

#### 2.1.2 Baseline measures

At baseline, participants reported their knee symptoms over the last seven days using VAS (Visual Analogue scale) knee pain (0–100 ​mm) [20], WOMAC (Western Ontario and McMasters Universities Osteoarthritis Index) knee pain (0–500 ​mm), WOMAC knee function (0–1700 ​mm) and WOMAC knee stiffness (0–200 ​mm) (lower is better) [21]. Physical performance was measured using physical performance tests that are recommended by OARSI [22], including the 30 ​s Chair Stand Test, 40 ​m Fast-paced Walk Test (m/s), 6-min walk test (higher scores are better), the Timed up and Go test and Stair Climb Test (lower scores are better), and by an Isometric leg strength test (predominantly quadriceps and hip extensors) for both legs simultaneously in kilogram (kg) by dynamometry (TTM Muscular Meter Tokyo, Japan). Physical activity was assessed by ActiGraph® wGTX3-BT (Firmware 1.9.2) activity monitors (ActiGraph LLC, Fort Walton Beach, FL, USA), which was waist worn for one week during screening. The accelerometer data were processed using settings as recommended by Migueles et al. [23]. Health related quality of life and utility was assessed using the Assessment of Quality of Life (AQoL-8D) [24] (0–1) and the EuroQol 5-Dimension 5-Level (EQ-5D-5L) [25] (0–1) questionnaires (1 ​= ​full health). Depression was assessed using the Patient Health Questionnaire (PHQ-9) [26] (0-27) (lower is better). At screening the use of medications was recorded by questionnaire and confirmed by the research assistant. No restrictions were made regarding analgesic medications. Participants were asked to keep medications as stable as possible but if a participant required an increase in analgesics this was permitted and recorded during the study.

#### 2.1.3 Additional measures

At screening information on participant's sex, date of birth, marital and educational status was collected. Weight was measured to the nearest 0.1 ​kg (with shoes, socks, and bulky clothing removed) using a single pair of electronic scales (Seca Delta Model 707). Height was measured to the nearest 0.1 ​cm (with shoes and socks removed) using a stadiometer. Body mass index (BMI) was calculated as kg/m^2^. History of previous joint injury and surgery was collected via a questionnaire. A standing semi-flexed X-ray of the study knee was taken from which knee alignment and radiographic knee OA were measured. Static knee alignment was derived from the anatomic axis based upon the methods of Moreland et al. [27]. Radiographic knee OA severity was measured by consensus with two readers (GJ, SD) utilizing the OARSI atlas to grade osteophytes and joint space narrowing (JSN) [28]. Intra-observer reproducibility was assessed in 20 participants. The ICC (2-way mixed-effects model) was 0.95 for knee alignment, 0.84 for osteophytes, and 0.93 for JSN.

### 2.2 Pain measurement and operational definitions

An 11-point Numeric Rating Scale (NRS from 0 to 10) [29] was used at each class to measure “what is your pain right now?” These pain levels were verbally reported by participants to their supervisor before the warm up at each class (pre-exercise pain) and shortly after completing the cool down at each class (post-exercise pain). The attending supervisor documented the pain values in the database.

*Acute movement-evoked pain* refers to a change in pain that is triggered during physical activity [2] (i.e., movement such as walking, cycling, sports, etc) [1,3]. In the context of the current study, it pertains to the walk class. The term ‘acute’ emphasizes that the pain change is measured in real-time during class attendance. AMEP can either be an increase in pain (i.e., flare), pain relief, or no change.

*First objective:* AMEP was calculated by subtracting the pre-from the post-exercise pain. In addition, AMEP at the first walk, the last walk in week 12 and the last walk in week 24 was categorized to indicate the number of participants with an AMEP flare (positive value), no change (0) or an AMEP relief (negative value).

*The second objective* was to compare participants with either higher or lower attendance rates by analyzing their (i) baseline physical performance and strength test results and (ii) number of participants with an AMEP and average AMEP (continuous) during the first class and in the three subsequent classes (class 2, 3, and 4).

### 2.3 Statistical analyses

The analyses were performed using Stata (version 16; StataCorp, College Station, TX, USA). The characteristics of the participants are given as mean values ​± ​standard deviation (SD) for continuous variables and as number and proportions for categorical variables. Descriptive statistics were used to summarize means and SD of pre-exercise pain and AMEP, and the number and proportion of participants with an AMEP flare, relief, or no change. For the continuous analyses, mixed-effects models with random intercepts by participant ID and maximum likelihood were used to investigate the slope in pre-exercise pain and AMEP, defined as a trajectory [7,8]. The improvement per class over any time interval was estimated by calculating the predicted value of the outcome for the session closest to the mid-point of a specified time interval, and subtracting it from the predicted value of the outcome for the next (higher) session. Tests were conducted to investigate assumptions for mixed-effects models. It was anticipated that pain, both pre-exercise and AMEP, would likely follow a trajectory of decreasing change from early to late follow-up. Both models were tested with a quadratic fit to account for this pattern. Likelihood ratio testing confirmed that a quadratic fit was a significantly better fit to the data. For the second objective, a comparison of means was conducted to compare baseline differences in physical performance and strength between participants with either a higher or lower attendance rate.

## 3 Results

Twenty-four participants with clinically diagnosed knee OA started the 24-week walking intervention. At baseline, the participants had a mean age of 65.1 years (SD 8.9), n ​= ​15 (62.5%) were female, and mean BMI was 33.6 ​kg/m^2^ (SD 4.9) (Table 2). Over the 24-week walking program, attendance of the 48 scheduled supervised walk classes was 60.8% and completion of the 24 unsupervised walk sessions was 88.4%. During the intervention period, n ​= ​7 (29%) participants withdrew. All withdrawals occurred within the first 12 weeks. Self-reported reasons for withdrawal included preoccupied with work responsibilities and cannot cope with walks as well [n ​= ​1], increased overall knee pain, attributed to their work [n ​= ​1], unrelated health problems [n ​= ​2], knee injury [n ​= ​2], and going away [n ​= ​1]. The highest AMEP flare reported at the last attended class by a participant who withdrew was NRS 2.

**Table 2:** Baseline characteristics of participants.

Baseline characteristics	N	
Age, years: mean (SD)	24	65.1 (8.9)
Women: n (%)	24	15 (62.5)
BMI, kg/m^2^: mean (SD)	24	33.6 (4.9)
Waist/hip ratio: mean (SD)	24	0.9 (0.1)
**Symptoms**^a^		
VAS pain (0–100 ​mm): mean (SD)	24	55.3 (17.3)
WOMAC pain (0–500 ​mm): mean (SD)	24	250.0 (78.7)
WOMAC function (0–1700 ​mm): mean (SD)	24	915.8 (232.9)
WOMAC stiffness (0–200 ​mm): mean (SD)	24	107.4 (35.5)
**Performance and strength**		
30 ​s Chair Stand Test^b^: score mean (SD)	24	11.0 (1.9)
40 ​m Fast-paced Walk Test (m/s)^c^: mean (SD)	24	29.7 (4.7)
6 ​min Walk Test (m)^d^: score mean (SD)	24	478.5 (91.7)
Stair Climb Test (s)^e^: score mean (SD)	24	20.2 (9.1)
Timed up and Go (s)^e^: score mean (SD)	24	9.5 (1.5)
Leg strength (kg)^f^: mean (SD)	24	71.0 (8.8)
**Questionnaires**		
PHQ-9 score (0-27)^g^: mean (SD)	24	4.4 (4.5)
EQ-5D-5L index value (0–1)^h^: mean (SD)	24	0.7 (0.0)
Aqol-8D utility score (0–1)^i^: mean (SD)	24	0.8 (0.1)
**Medication usage**		
Paracetamol usage: n (%)	24	8 (33.3)
Dose: mg: mean (SD)	8	1039.0 (330.0)
COX-2 inhibitor usage: n (%)	24	0 (0)
NSAIDS usage: n (%)	24	9 (37.5)
**Accelerometer measures**^j^		
Sedentary minutes per week: mean (SD)	23	3830.6 (851.6)
Light PA minutes per week: mean (SD)	23	1876.1 (485.0)
MVPA minutes per week: mean (SD)	23	123.1 (97.9)
**Radiographic measurements**		
Alignment^k^: n (%)	23	
Normal alignment		3 (13.0)
Genu varus		16 (70.0)
Genu valgus		4 (17.4)
Osteophytes (0-12): mean (SD)^l^	23	3.1 (4.2)
Radiographic medial JSN^l^: n (%)	23	
Grade 0		8 (34.8)
Grade 1		4 (17.4)
Grade 2		5 (21.7)
Grade 3		6 (26.1)
Radiographic lateral JSN^l^: n (%)	23	
Grade 0		16 (60.6)
Grade 1		2 (8.7)
Grade 2		2 (8.7)
Grade 3		3 (13)

Statistics are presented as mean and standard deviation (SD) for continuous variables or n (proportion) for categorical variables. Abbreviations: BMI ​= ​body mass index; COX ​= ​cyclooxygenase; NSAID ​= ​non-steroidal anti-inflammatory drugs.

a Higher score in Visual Analogue Scale (VAS) pain and WOMAC, Western Ontario and McMaster Universities Arthritis Index (WOMAC) indicates a more severe symptom.

b Number of stances made within 30 ​s.

c Walking speed in m/s.

d Meters walked within 6 ​min.

e Time in seconds.

f Force in KG.

g Patient Health Questionnaire (PHQ-9), a depression module in which a lower score is better.

h EURoQol-5 dimensions-5 levels (EQ5D -5L), index values were calculated using the UK data set, a score closer to 1 is better.

i Assessment of Quality of Life (AQoL), utility scores were obtained by AQoL utility formulae, a score closer to 1 is better.

j PA accelerometer, change in physical activity (PA) objectively measured by accelerometer including minutes in moderate to vigorous physical activity (MVPA).

k Anatomic knee alignment based upon methods of Moreland et al. [28].

l Mean cumulative grade of osteophytes and grade of joint space narrowing (JSN) determined utilizing the OARSI atlas [30], a lower grade is better.

### 3.1 Pre-exercise pain before starting the walk

**Fig. 1:**
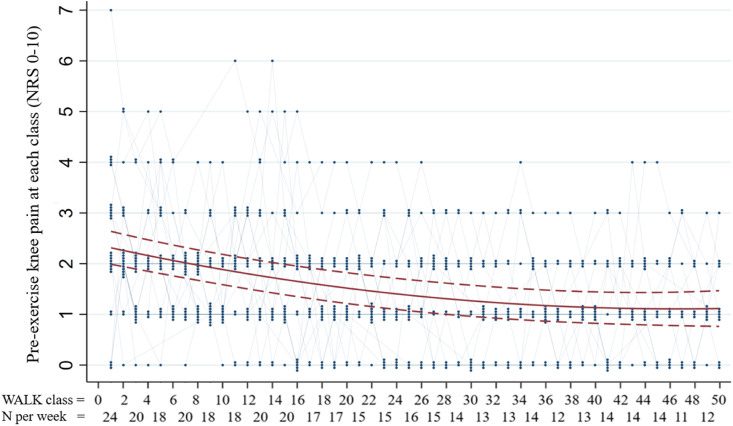
Fitted trajectory of pre-exercise knee pain over time (consecutive walk classes) derived from a mixed-effects model (red line). The y axis indicates the NRS (0-10) pre-exercise pain. The x axis shows each cumulative walk class (two walks ​= ​1 week) and the number of participants who attended that week (N). The blue dots illustrate the pre-exercise values that were reported at each class with the number of dots indicating the number of participants who reported the same pain value.

At the first class, pre-exercise pain ranged from NRS 0 to 7 (Fig. 1). After 8 weeks (16 classes) of intervention, no pre-exercise pain higher than NRS ​= ​4 was observed. The quadratic model fit is shown in Fig. 1. Predicted values and slopes from the quadratic model were as followed; average pre-exercise pain was estimated to improve by NRS 1.20 (95% CI −1.41 to −0.99) over the 24-week walking program. The improvement in pre-exercise pain per class was largest at the start of the intervention (Fig. 1). The estimated improvement per class was NRS 0.05 (95% CI: 0.06 to −0.03) during the initial 8 weeks but smaller at NRS 0.02 (95% CI: −0.03 to −0.02) during the next 12 weeks of the program, and reached a plateau around the 20-week mark (Fig. 1).

### 3.2 Acute movement evoked pain (AMEP)

**Fig. 2:**
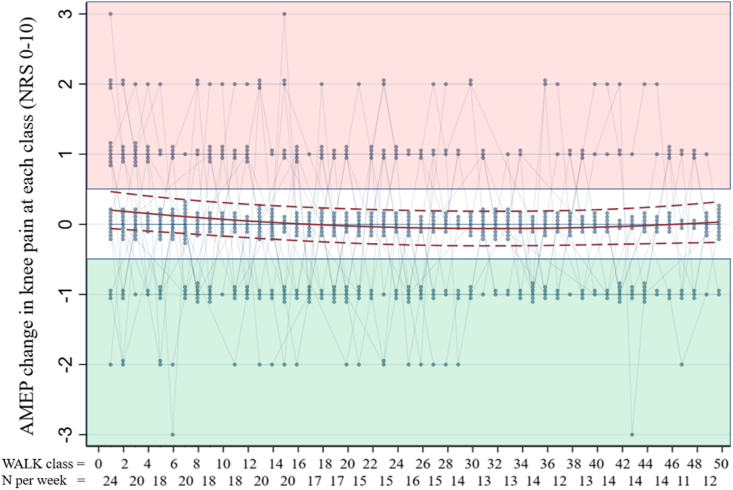
Fitted trajectory of acute movement-evoked pain (AMEP) change over time (consecutive walk classes), derived from a mixed-effects model (red line). The y axis indicates the AMEP change in NRS (0-10) knee pain at each walk class. The x axis shows each cumulative walk class (two walk classes ​= ​1 week) and the number of participants who attended that week (N). The blue dots illustrate the AMEP change values at each class, with the number of dots indicating the number of participants who had the same AMEP change. Positive values (in the red section) indicate an AMEP flare and negative values (in the green section) an AMEP relief.

At the first class, 45.8% [n ​= ​11] of participants reported an AMEP flare, and this decreased to 23.5% [n ​= ​4] at week 12 and 24. (Table 3). The quadratic model fit is shown in Fig. 2. Predicted values and slopes from the quadratic model for AMEP were as followed: mean AMEP improved by NRS 0.19 (95% CI −0.38 to −0.004) over the 24-week walking program. The estimated improvement in AMEP per class was NRS 0.02 (95% CI: −0.03 to ​< ​−0.01) during the first week but smaller at NRS 0.01 (95% CI: 0.02 to ​< ​−0.01) during the initial 8 weeks of the program, and reached a plateau approximately at the 12-week mark (Fig. 2).

**Table 3:** Proportion of participants who had an AMEP flare, no change or an AMEP relief at the first walk, week 12 and week 24 weeks.

AMEP change	First walk N ​= ​24	Week 12 N ​= ​17	Week 24 N ​= ​17
AMEP flare, N (%)	11 (45.8)	4 (23.5)	4 (23.5)
No change, N (%)	9 (37.5)	8 (47.1)	8 (47.1)
AMEP relief, N (%)	4 (16.7)	5 (29.4)	5 (29.4)

Week 12: Based on acute pain change at walk 24, otherwise walk 25.

### 3.3 Differences between participants with either a higher or lower attendance rate

Of the 24 participants who started the intervention, n ​= ​13 (54%) attended 70% or more of the 48 scheduled supervised walking classes. Eleven participants attended less than 70% of the 48 classes, of whom n ​= ​7 withdrew completely from the study.

The participants with lower attendance had at baseline poorer physical performance and leg strength on all tests, including the Six Minute Walk Test (418.8 ​m (95% CI 367.8 to 469.8) vs 529.0 ​m (95% CI 485.1 to 572.9)), the 30 ​s Chair Stand Test (10 stances (95% CI 8.8 to 11.2) vs 12.0 stances (95% CI 10.9 to 12.9)), Fast Past Walk Test (1.23 ​m/s (95% CI 1.1 to 1.3) vs 1.51 ​m/s (95% CI 1.4 to 1.6)), and the Timed Up and Go Test (10.2 ​s (95% CI 9.1 to 11.3) vs 9.0 ​s (95% CI 8.2 to 9.8)), and leg strength (59.5 ​Kg (95% CI 36.6 to 82.4) to 80.7 ​Kg (95% CI 51.2 to 110.1)).

Furthermore, at baseline n ​= ​6 (60%) participants with lower attendance and n ​= ​7 (54%) of participants with higher attendance were taking pain medication. After 12 weeks of intervention, one participant with higher attendance stopped using NSAID and one participant with lower class attendance increased paracetamol usage. No changes in analgesic medication were reported at 24 weeks.

At the first class, the largest proportion of participants with an AMEP flare was observed. During this first class, participants with lower attendance reported an average AMEP of NRS 0.18 (SD 0.9), with 36% (4 out of 11) experiencing a flare. This was less compared to participants with higher attendance, who reported an average AMEP change of NRS 0.69 (SD 1.6), with 54% (7 out of 13) experiencing a flare.

In the following classes over the first two weeks (comprising an average of classes 2, 3, and 4), the average AMEP for participants with higher attendance decreased to NRS 0.11 (SD 1.2) [n ​= ​13], while the average AMEP for participants with lower attendance increased to NRS 0.44 (SD 0.9) [n ​= ​11].

## 4 Discussion

The results from this exploratory study showed that over 24-weeks of the WALK program, individuals with knee OA improved in pre-exercise knee pain over the initial 20 weeks and in AMEP over the initial 12 weeks. The number of participants experiencing AMEP flares decreased by almost 50% in the first 12 weeks of the WALK program and remained consistent during the last 12-weeks of the program. Furthermore, we showed that participants with lower attendance scored poorer on all physical performance tests and experienced a slight increase in AMEP during the first two weeks of the program.

After 24 weeks of the WALK program, average pre-exercise pain had improved by NRS 1.2, which exceeds the minimal clinically important improvement (MCII ​= ​1 NRS-points) found in people with symptomatic knee OA [31]. During the first 8 weeks, the estimated improvement in pre-exercise pain per class (−0.05 (95% CI: 0.06 to −0.03) was similar to the improvement per neuromuscular exercise session measured by Sandal et al. [8], during their 8 week NEMEX program (NRS -0.04 (95% CI −0.02 to −0.05)). Albeit the WALK participants had lower pre-exercise pain at the first class, which enabled less scope for improvement.

To the best of our knowledge, this study is the first to investigate a trajectory in AMEP over more than 12 weeks in people with knee OA. At the first walking class, about half of the participants experienced an AMEP flare and this reduced to one in four participants at week 12. From weeks 12–24, AMEP remained consistent, demonstrating a quadratic relationship. In line with the findings of the current study, Primeau et al. [7] also observed a decrease in AMEP flares following a quadratic trajectory, in a similar population undergoing a 12-week neuromuscular exercise program. Interestingly, they showed that the first neuromuscular session resulted in an average AMEP flare up of approximately 1 NRS unit [7], which was more than twice as high as the average AMEP measured during the first walking class in our study. This suggests that aerobic walking may be less pain provocative at the first session compared to neuromuscular exercise. However, due to the heterogeneity in type of exercise and patient characteristics, the comparisons made with the findings of Sandal et al. [8] and Primeau et al. [7] need to be interpreted with caution. Furthermore, the extent to which improvements could be observed in this study may have been limited by the low average flare in AMEP at the first walking class. In addition, pre-exercise pain was used to calculate AMEP at each class. Therefore, the improvement in pre-exercise pain over the course of the program reduced the potential for observing improvements in AMEP. Moreover, while walking intensity remained constant, participants’ actual physical performance levels increased over time [15]. It is plausible that these improvements could have influenced the observed trajectory in AMEP.

A large body of literature addresses the reasons why some individuals with knee OA may avoid exercise, including concerns about exercise-induced pain (i.e., AMEP) and adequacy of the exercise to physical capabilities [14,32,33]. Participants who attended less classes were characterized by poorer physical performance at baseline but the difference in AMEP between participants with higher and lower attendance was less clear. In the first class, participants with higher attendance showed a higher average AMEP compared to participants with a lower attendance. However, in subsequent classes (2, 3, 4), the prior exhibited a decline in AMEP, while participants with lower attendance showed a slight increase. These results, although based on a limited number of observations, might suggest that AMEP in the first class may not influence future class attendance, but a higher average of AMEP over the rest of the first 2 weeks may be influential. These results generally align with the hypothesis put forth by Sandal et al. [8], that AMEP flares during the first two weeks of the program may affect attendance.

In both the current study and the study by Sandal et al. [8], participants did not receive any supplementary patient education alongside the exercise regimen. Nonetheless, educating patients about pain beliefs by supervisors has been recommended to improve adherence to exercise programs [34] and could still be considered as a way to enhance adherence in future studies on the WALK program. Furthermore, our study observed differences between participants with either lower or higher attendance in physical performance and strength. At baseline, participants with lower attendance walked on average 110 ​m less than participants with higher attendance in the Six Minute Walk test, which is quite substantial considering that the minimal clinically important difference for individuals with knee OA is 50 ​m [35]. Notably, average leg strength of participants with lower attendance was at baseline 26% lower compared to participants with higher attendance and nearly 40% lower compared to adults in a large Tasmanian cohort with comparable age and sex [36]. In a study by Sutton et al. (2022) [37], the views of individuals with knee OA on outdoor group walking were examined, finding that some participants may feel discouraged by others who are more physically fit and walk faster. This dynamic may have occurred in the present study as well, leading to reduced attendance. The formation of walking groups with a similar physical performance could be a potential solution to further improve attendance.

This study enabled us to frequently assess AMEP changes at supervised exercise classes over a relatively long (24-week) intervention period. There were certain limitations to this study. First, this is an exploratory study and results should be interpreted with caution because of the small sample size. A larger sample size increases the statistical power to detect genuine effects, but also reduces the likelihood of attributing chance findings as statistically significant results (reduced type I error) [38]. Second, the unavailability of a control group prevents us from attributing the observed changes in AMEP to the walking intervention, as regression-to-the-mean and contextual effects most likely also contributed. Third, there is no golden standard to define desirable attendance and a different cut-off could have influenced the results. Fourth, participants reported pain levels to their supervisor in the presence of other participants, which may have caused socially desirable answers. Last, a recent systematic literature review from Leemans et al. [10] on movement evoked pain in people with musculoskeletal conditions, suggested that future studies should include the measurement of movement-evoked pain that occurs while exercising and pain that is induced by standardized physical performance tests. Including these measurements could have improved the comparability of results between studies [10].

## 5 Conclusion

The WALK participants improved in pre-exercise pain and AMEP changes in the first 20 and 12 weeks, respectively. While the improvement in AMEP changes was modest, less participants experienced AMEP flares over time. The results of the current study may help clinicians provide more realistic expectations about changes in pain that participants may experience from a walking program. Additionally, an increased focus is warranted on participants who have poor baseline physical performance levels and experience an increase in AMEP during the first two weeks of walking, as this may contribute to lower attendance of supervised classes.

## Ethics statement

This study was approved by the Tasmanian Health and Medical Human Research Ethics Committee (H0017108).

## Funding

This trial is funded by Arthritis Australia, Arthritis and Osteoporosis Tasmania, and the University of Tasmania Better Health Theme research grant. Dr Callisaya, Antony, Jones, Hinman and Bennell are recipients of National Health and Medical Research Council of Australia (NHMRC) fellowship funding to support their salaries. Dr Aitken is the recipient of a NHMRC/Medical Research Future Fund (MRFF) fellowship to support her salary. Dr Scheepers is a recipient of the Farrel Family Postdoctoral Research Fellowship.

## Role of the funding source

Arthritis Australia, Arthritis and Osteoporosis Tasmania and the University of Tasmania Better Health Theme had no role in the design and conduct of the study; collection, management, analysis, and interpretation of the data; preparation, review, or approval of the manuscript; and decision to submit the manuscript for publication.

## Contributions

All authors were involved in drafting the article or revising it for important intellectual content. All authors have approved the final manuscript. Dr. Lieke Scheepers (lieke.scheepers@utas.edu.au) takes responsibility for the integrity of the work as a whole, from inception to finished article.

Conception and design: SJJD, SB, KB, RSH, MC, BA, TW, GJ, DA, LEJM.

Analysis and interpretation of data: SJJD, TW, PO, LB, DA, LEJM.

Drafting the article: SJJD, DA, LEJM.

Critical revision of the article for important intellectual content: SJJD, KB, RSH, MC, GJ, TW, PO, DA, LEJM.

Provision of study materials or patients: DA.

Statistical expertise: PO, LB.

Obtaining of funding: DA, MC, SB.

Administrative, technical or logistic support: SJJD, DA, KB, RSH, MC, IM, BA, TW, PO, LB, LEJM.

Collection and assembly of data: SJJD, IM, GJ, MC, TW, DA, LEJM.

## Transparency declaration statement

The lead authors affirm that this manuscript is an honest, accurate, and transparent account of the study being reported; that no important aspects of the study have been omitted; and that any discrepancies from the study as planned (and, if relevant, registered) have been explained.

## Data availability statement

The data generated from this study will not be deposited in a public repository due to privacy and consent restrictions. De-identified data can be made available from the corresponding author on reasonable request, subject to a data sharing agreement.

## Consent to participate

All participants provided written informed consent.

## Statements and declarations

Arthritis Australia, Arthritis and Osteoporosis Tasmania and the University of Tasmania Better Health Theme had no role in the design and conduct of the study; collection, management, analysis, and interpretation of the data; preparation, review, or approval of the manuscript; and decision to submit the manuscript for publication. The authors do not have any conflicts of interest relevant to this study.

## Conflicts of interest

The authors do not have any conflicts of interest relevant to this study.
